# Prognostic Value of Temporary Pacemaker Insertion in Patients with Acute Myocardial Infarction in the Era of Percutaneous Coronary Revascularization

**DOI:** 10.31083/j.rcm2406179

**Published:** 2023-06-19

**Authors:** Peng Wang, Shidong Wang, Zhimin Liu, Lei Song, Bo Xu, Kefei Dou, Yongjian Wu, Shubin Qiao, Runlin Gao, Gang Zhao, Mi Huang, Xuemei Hu, Hao Wang, Xuelian Xu, Yuejin Yang

**Affiliations:** ^1^Department of Cardiology, Fuwai Hospital, National Center for Cardiovascular Diseases, Chinese Academy of Medical Sciences and Peking Union Medical College, 100037 Beijing, China; ^2^Department of Cardiology, Linyi People’s Hospital of Shandong Province, 251500 Dezhou, Shandong, China; ^3^Department of Cardiology, University-Town Hospital of Chongqing Medical University, 401331 Chongqing, China; ^4^Department of Cardiovascular Medicine, Chongqing Emergency Medical Center, Chongqing University Center Hospital, 400014 Chongqing, China

**Keywords:** acute myocardial infarction (AMI), arrhythmia, temporary pacemaker (tPM), coronary revascularization

## Abstract

**Background:**

Patients with acute myocardial infarction (AMI) complicated with arrhythmia are not uncommon. Insertion of temporary pacemakers (tPMs) in patients with arrythmia during acute myocardial infarction (AMI) is imperative support therapy. Arrhythmias include high-degree atrioventricular block (AVB), sinus arrest/bradycardia, and ventricular arrythmia storm. To date, no study has evaluated the prognosis of tPMs in patients with AMI complicated with arrhythmia. Especially in the era of thrombolysis or emergency percutaneous coronary intervention (PCI) for coronary artery revascularization, our study was designed to investigate the value of tPMs implantation in cases of AMI complicated with various arrhythmias.

**Methods:**

From January 2009 to January 2019, 35,394 patients with AMI, including 62.0% (21,935) with ST-segment elevation myocardial infarction (STEMI) and 38.0% (13,459) with non-ST-segment elevation myocardial infarction (NSTEMI) in four hospitals, were reviewed. A total of 552 patients with AMI associated with arrythmia were included in the cohort. Among the 552 patients, there were 139 patients with tPM insertions. The incidence trend of myocardial infarction complicated with various arrhythmias in the past 10 years was analysed, and the clinical characteristics, in-hospital mortality, postdischarge mortality, composite endpoints of modality, and independent risk factors were compared in patients with and without tPM in the era of coronary artery revascularization.

**Results:**

In patients with AMI-associated arrythmia, high-degree AVB was the major cause of tPM insertion (*p* = 0.045). In the past 10 years, the number of patients with high-degree AVB, tPM implantation, ventricular arrythmia storm, and in-hospital mortality has decreased year by year in the era of coronary artery revascularization. In the tPM group, the culprit vessel was the left main artery, and cardiogenic shock, acute renal injury and high brain natriuretic peptide (BNP) levels were independent risk factors for patients with AMI complicated with arrhythmia. The in-hospital mortality in the tPM group was higher than that in the non-tPM group. The patients with tPM insertion showed better postdischarge survival than patients without tPM insertion.

**Conclusions:**

In the era of emergency thrombolysis or PCI, coronary revascularization can ameliorate the prognosis of patients with AMI complicated with various arrhythmias. Temporary pacemaker insertion in patients with AMI complicated with arrhythmia can reduce the postdischarge mortality of these patients.

## 1. Background

Insertion of transvenous temporary pacemakers (tPMs) in patients with bradycardiac arrythmia during acute myocardial infarction (AMI) is imperative support therapy, which was widely studied in the era before the generalization of intravenous fibrinolysis and primary percutaneous coronary revascularization. The majority of tPM insertions in patients with AMI were due to high-degree or complete atrioventricular block (AVB). In the era before coronary revascularization, the average in-hospital mortality for inferior wall AMI without AVB was 9%, compared with 23% in patients with high-degree AVB and 29% in patients with third-degree AVB. Recently, a large retrospective analysis from the Global Registry of Acute Coronary Events reported that high-degree AVB had a strong association with in-hospital but not late mortality, while tPMs therapy was not associated with improved in-hospital survival [1].

Compared with the 1990s, in the last 10 years, we observed a perceptible decrease in the incidence of temporary pacemaker therapy in patients with AMI in clinical practice. The aim of this study was to determine the incidence of tPM insertion, death and permanent PM implantation associated with tPM insertion and risk factors associated with death in patients with tPM during either ST-segment elevation myocardial infarction (STEMI) or non-ST-segment elevation myocardial infarction (NSTEMI) in the era of fibrinolysis and primary percutaneous coronary revascularization.

## 2. Methods

This is a retrospective multicentre study that enrolled patients with AMI at 4 hospitals (Fuwai Hospital, National Center for Cardiovascular Diseases, Chinese Academy of Medical Sciences and Peking Union Medical College, Linyi People’s Hospital of Shandong Province, University-Town Hospital of Chongqing Medical University, Chongqing University Center Hospital) in China between 2009 and 2019. Eligible patients were ≥18 years old with a presumptive diagnosis of AMI and ≥1 of the following examination findings: electrocardiography (ECG) changes consistent with AMI, abnormal cardiac biomarkers, or history of coronary artery disease. Patients were excluded if AMI was precipitated by noncardiovascular comorbidities, including gastrointestinal bleeding, trauma advanced neoplasms or an operation. This trial has been supported by the Medical Ethics Committee of University-Town Hospital of Chongqing Medical University (LL-202014). All participating patients signed informed consent forms.

ECGs were interpreted at each enrolling centre by 2 experienced cardiologists and not centrally adjudicated. ST-segment elevation of ECGs was defined as ≥1 mm ST-segment elevation in two contiguous leads or new happening left bundle branch block. STEMI was diagnosed as new ST elevation or new happening left bundle branch block accompanied by ≥1 positive cardiac biomarker confirming myocardium necrosis. NSTEMI was diagnosed when ≥1 positive cardiac biomarker confirming myocardium necrosis was present without new ST-segment elevation. High-degree AVB was diagnosed as the presence of either Mobitz II second-degree AVB or third-degree AVB.

Indication for transvenous tPMs insertion was defined as: (1) new high-degree AVB due to acute myocardial ischaemia or complications during percutaneous coronary intervention (PCI) (such as no reflow, or iatrogenic coronary dissection); (2) new sinus arrest >3 s or sinus bradycardia or escape rhythm with a cardiac rate <35 bpm; or (3) ventricular arrythmia storm with bradycardic arrythmia with a cardiac rate <50 bpm. The transvenous tPM catheter was inserted into the right ventricular apex via right jugular or femoral routes within a 5F short sheath.

Standardized case report forms were completed by study physicians to document patient data, clinical history, clinical manifestation, medication use (before and during hospitalization), in-hospital treatment (medical and invasive therapies), and in-hospital clinical events (death, permanent pacemaker, cardiogenic shock, sustained ventricular tachycardia, or stroke). The primary endpoints were the occurrence of in-hospital and postdischarge death. Secondary endpoints were in-hospital and postdischarge permanent pacemaker implantations. The follow-up consisted of a clinical visit every 6 months and contacts by telephone every 12 months to identify vital status and new clinical events.

### Statistical Analysis

Patients with AMI and myocardial ischaemia-associated arrythmia (high-degree arrythmia, sinus arrest or bradycardia, or ventricular shock with bradycardia occurring at any time during AMI) were divided into two groups: those with tPM insertion and those without tPM insertion at any time during AMI. Mean ± SD or medians with 25th and 75th percentiles were calculated for continuous variables, and absolute and relative frequencies were measured for categorical variables. For continuous variables, differences between groups were analysed for statistical significance by the 2-tailed *t *test or Mann-Whitney U test. The chi-square test was applied to compare differences between categorical variables. Univariate and multiple logistic regression analyses were used to estimate the odds associated with different clinical factors and in-hospital mortality within this group of patients. Odds ratios (ORs) and 95% confidence intervals (CIs) were calculated. All tests of significance were 2-tailed. A *p* value of 0.05 was considered statistically significant. All analyses were conducted using the SAS software package, version 9.2 (SAS Institute, Cary, NC, USA).

## 3. Results

### 3.1 Baseline Characteristics

**Fig. 1.**
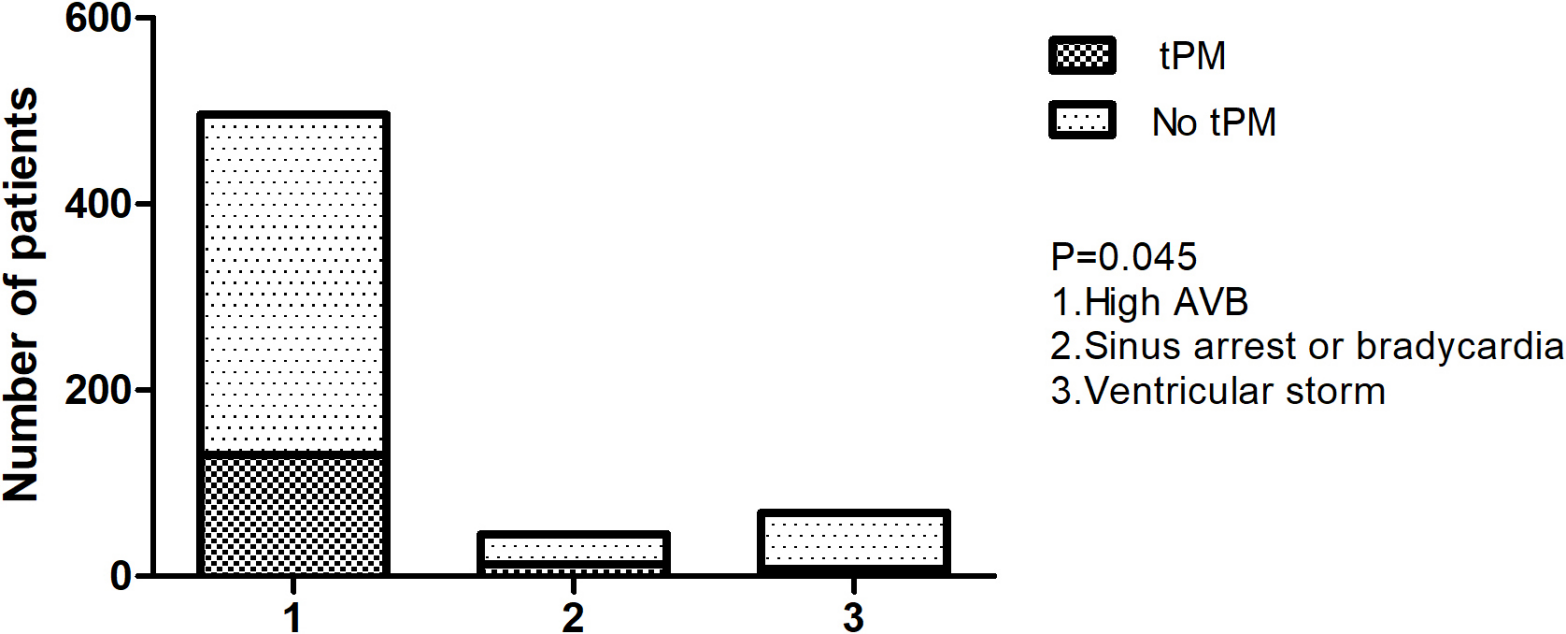
**Temporary pacemaker in different arrythmias due to AMI**. tPM, temporary pacemaker; AVB, atrioventricular block; AMI, acute myocardial infarction.

A total of 35,394 patients with AMI hospitalized in 4 hospitals (Fuwai Hospital, Linyi People’s Hospital of Shandong Province, University-Town Hospital of Chongqing Medical University, Chongqing University Center Hospital) between 2009 and 2019 were continuously included: 62.0% (21,935) STEMI and 38.0% (13,459) NSTEMI. In the past 10 years, the incidence of AMI and the number of PCIs have increasing. Among the patients with STEMI, 10,723 (30.3%) patients with acute inferior infarction were identified. The overall tPM insertion rate of the cohort was 0.39% (139/35,394). In patients with cardiac ischaemia-associated arrythmias (552/35,394, 1.6%), high-degree AVB was the major cause of tPM insertion (*p* = 0.045, Fig. 1), and the rate of tPM insertion showed no significant difference in patients with STEMI compared with that in patients with NSTEMI (0.14% vs. 0.15%, *p* = 0.331). The median time of tPM usage was 103 (48–192) hours, and no significant difference was recorded between the STEMI and NSTEMI cohorts (*p* = 0.103).

**Fig. 2.**
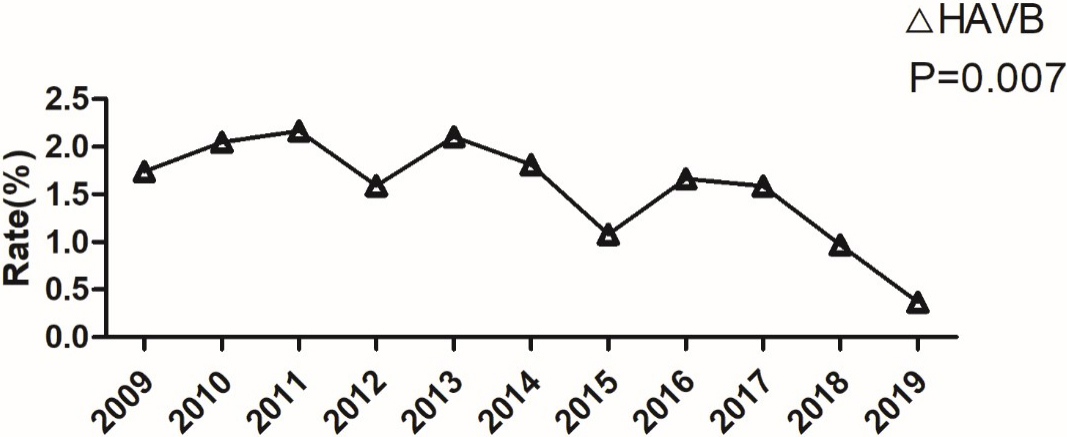
**Incidence of acute myocardial infarction complicated with high-degree**. HAVB, high-degree atrioventricular block.

**Fig. 3.**
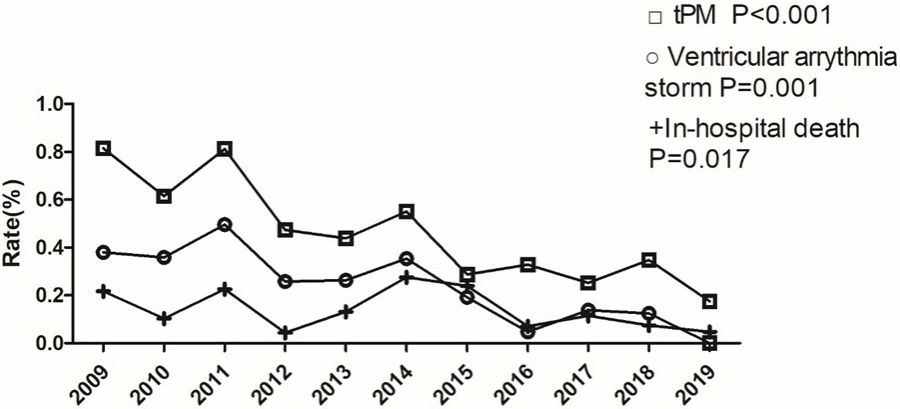
**Incidence of acute myocardial infarction complicated with ventricular arrythmia storm, temporary pacemaker implantation and in-hospital mortality in the last 10 years**. tPM, temporary pacemaker.

This study observed the change trend of patients with AMI and high-degree AVB, sinus arrest/bradycardia, ventricular arrythmia storm, the proportion of temporary and permanent pacemaker insertion and in-hospital mortality in the last 10 years. AMI with high-degree AVB (linear trend = –0.1; *p* = 0.007, Fig. 2), combined with ventricular arrythmia storm (linear trend = –0.1%; *p* = 0.001, Fig. 3), proportion of tPM implantation (linear trend = –0.1%; *p* < 0.001, Fig. 3), and in-hospital mortality (linear trend = –0.1%; *p* = 0.017, Fig. 3), showed a decreasing trend year by year. Patients with AMI complicated with sinus arrest/bradycardia (HR <30 bpm) (linear trend = –0.1; *p *= 0.214), the proportion of permanent pacemaker implantation (linear trend = –0.1; *p* = 0.087), and the downwards trend year by year were not statistically significant.

The baseline characteristics of 552 patients are summarized in Table 1. Among 552 patients with AMI with arrythmia, 139 (25.2%) of them received tPM implantation. There were no significant differences in hypertension, diabetes, peripheral vascular disease, stroke, myocardial infarction, percutaneous coronary intervention, coronary artery bypass graft, heart failure, adenosine diphosphate (ADP) receptor blocker, β-blocker, angiotensin-converting enzyme inhibitor (ACEI), angiotensin receptor blocker (ARB), blood pressure, Killip class, ST-segment elevation, or left bundle branch block between tPM and non-tPM groups.

**Table 1.** **Baseline demographic and clinical characteristics at presentation and in-hospital invasive and medical therapies in patients with bradyarrhythmia or ventricular arrythmia storm due to AMI with or without temporary pacemaker insertion**.

			With temporary pacemaker (n = 139)	Without temporary pacemaker (n = 413)	*p-*value
Age			66.5 ± 11.8	63.8 ± 12.2	0.022
Male (%)			92 (66.2)	348 (84.3)	0.001
Medical history					
	Hypertension		90 (64.7)	270 (65.4)	0.812
	Diabetes		57 (41.0)	166 (40.2)	0.866
	Dyslipidaemia		91 (65.5)	312 (75.5)	0.021
	Peripheral vascular disease		11 (7.9)	42 (10.2)	0.435
	Stroke/TIA		25 (18.0)	76 (18.4)	0.913
	Myocardial infarction		12 (8.6)	63 (15.3)	0.050
	Percutaneous coronary intervention		7 (5.0)	52 (12.6)	0.913
	Coronary artery bypass graft surgery		5 (3.6)	23 (5.6)	0.203
	Heart failure		10 (7.2)	49 (11.9)	0.123
	Prehospital medication				
		Aspirin	62 (0.45)	290 (0.7)	0.001
		ADP receptor blocker	58 (0.42)	188 (0.46)	0.436
		β-blocker	27 (0.19)	107 (0.26)	0.123
		Statin	51 (0.37)	221 (0.54)	0.001
		ACEI	29 (0.21)	95 (0.23)	0.512
		ARB	28 (0.2)	89 (0.22)	0.726
Clinical presentation					
	Systolic blood pressure (mmHg)		117.5 ± 25.5	119.2 ± 21.3	0.428
	Killip class				
		I	83 (0.6)	284 (0.69)	0.071
		II	32 (0.23)	70 (0.17)	0.111
		III	7 (0.05)	18 (0.04)	0.740
		IV	17 (0.44)	41 (0.1)	0.444
	Arrythmia due to AMI				
		High-degree AVB	130 (0.94)	366 (0.89)	0.098
		Sinus arrest	13 (0.09)	32 (0.08)	0.550
		Ventricular arrythmia storm	8 (0.06)	60 (0.15)	0.006
		ST-segment elevation	121 (0.87)	341 (0.83)	0.216
		ST-segment depression	17 (0.12)	65 (0.16)	0.223
		Left bundle branch block	5 (0.036)	7 (0.017)	0.276
	LVEDD (mm)		48.5 ± 7.1	50.4 ± 6.8	0.005
	LVEF (%)		51.7 ± 8.9	53.9 ± 9.7	0.021

ADP, adenosine diphosphate; AMI, acute myocardial infarction; TIA, transient ischaemic attack; ACEI, angiotensin-converting enzyme inhibitor; ARB, angiotensin receptor blocker; AVB, atrioventricular block; LVEDD, left ventricular end diastolic diameter; LVEF, left ventricular ejection fraction.

However, there were significant differences in age, male sex, dyslipidemia, aspirin, statin use, ventricular arrythmia storm, left ventricular end diastolic diameter (LVEDD) (mm), and left ventricular ejection fraction (LVEF) (%). Older people, females tended to be more prevalent in the tPM group.

**Fig. 4.**
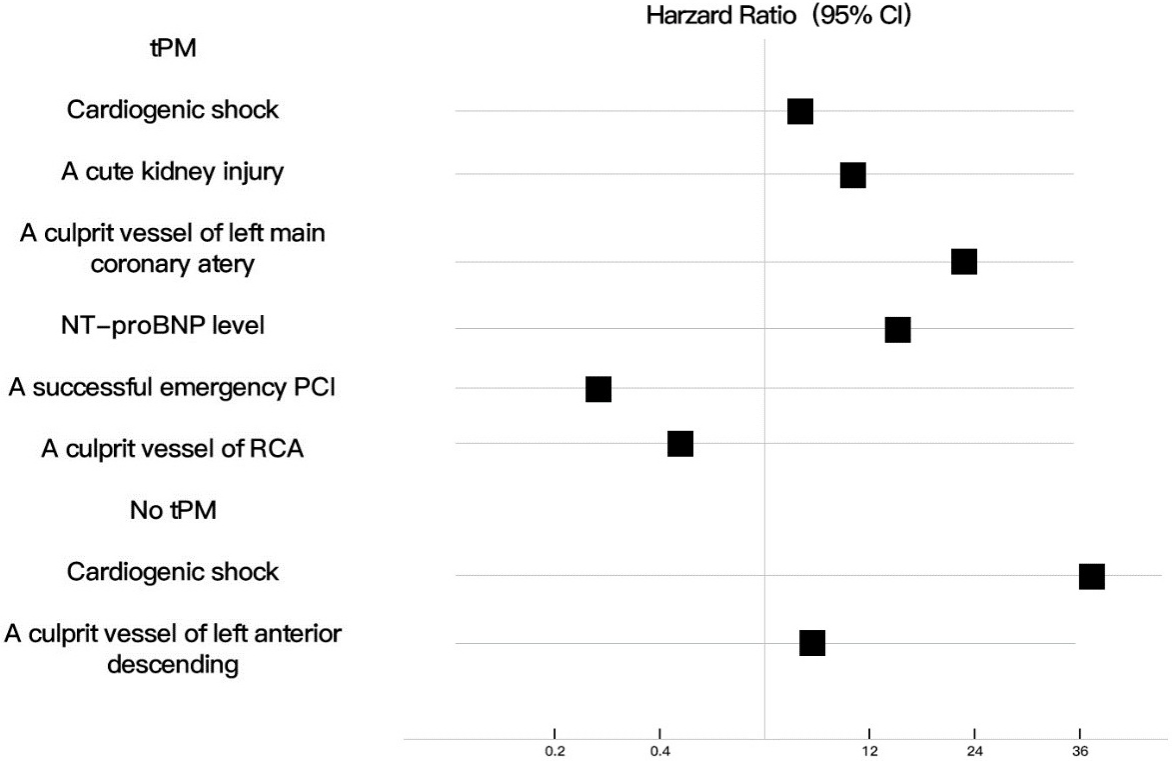
**Independent risk factors in patients with or without tPM insertion**. tPM, temporary pacemaker; NT proBNP, N-terminal pro-brain natriuretic peptide; PCI; percutaneous coronary intervention; RCA, right coronary artery.

Specifically, for patients with tPM insertion during AMI, cardiogenic shock (*p* = 0.044, 95% CI, HR = 4.384), acute kidney injury (*p* = 0.019, 95% CI, HR = 11.9), left main coronary artery as the culprit vessel (*p <* 0.001, 95% CI, HR = 23.4) and high N-terminal pro-brain natriuretic peptide (NT-proBNP) level (*p* = 0.002, 95% CI, HR = 14.7) were associated with a higher risk of postdischarge death, whereas a successful emergency PCI (*p* = 0.009, 95% CI, HR = 0.489) and right coronary artery (RCA) as the culprit vessel (*p* = 0.001, 95% CI, HR = 0.27) were associated with a higher likelihood of postdischarge survival. Moreover, for patients without tPM insertion during AMI, cardiogenic shock (*p <* 0.001, 95% CI, HR = 36.5) and left anterior descending artery as the culprit vessel (*p *= 0.001, 95% CI, HR = 5.72) were associated with a higher risk of postdischarge death (Fig. 4). The positive association between permanent pacemaker implantation and in-hospital survival was further assessed by evaluating the association between permanent pacemaker implantation and survival after hospital discharge at 6 months.

Table 2 shows the clinical characteristics of in-hospital events, coronary lesions and culprit vessels in the two groups of patients with and without tPM implantation. Coronary artery PCI; coronary artery bypass grafting; myocardial reinfarction; heart failure; cardiogenic shock; persistent ventricular tachycardia; stroke; permanent pacemaker implantation; implantable cardioverter-defibrillator (ICD); acute renal injury; degree of coronary artery stenosis greater than 50% (left main trunk, anterior descending branch, circumflex branch, or right coronary artery); culprit vessels (left main, anterior descending branch, circumflex branch, or right coronary artery); thrombolysis in myocardial infarction (TIMI) blood flow of culprit vessels before coronary PCI (grade 0, grade 1, or grade 2); number of coronary arteries with stenosis greater than 50% [1, 2]; and being discharged on aspirin, statin, and ARB or ACEI drugs in the two groups of patients were not statistically different. In-hospital death in the tPM implantation group was significantly higher than that in the non-tPM implantation group (*p* = 0.017). The culprit vessels were right coronary artery (*p* = 0.015), TIMI blood flow of culprit vessels before coronary PCI was grade 3 (*p* = 0.011), the number of vessels with coronary stenosis greater than 50% was 3 (*p* = 0.023), and the number of patients discharged on a β-blocker (*p* = 0.001) in the non-tPM implantation group was significantly higher than that in the tPM implantation group.

**Table 2.** **In-hospital procedures and events, coronary lesions and culprit vessels, and discharge medications of patients with bradyarrhythmia or ventricular arrythmia storm due to AMI with or without temporary pacemaker insertion**.

			With tPM (n = 139)	Without tPM (n = 413)	*p*-value
In-hospital procedure (%)					
	Cardiac catheterization		95 (68.3)	346 (83.8)	0.001
	Percutaneous coronary intervention		90 (64.7)	316 (76.5)	0.210
	Percutaneous coronary intervention <24 hours		88 (64.0)	280 (67.8)	0.332
	Coronary artery bypass graft surgery		3 (2.2)	6 (1.5)	0.549
	Thrombolytic therapy		1 (0.7)	3 (0.7)	1.000
In-hospital events (%)					
	Myocardial re-infarction		1 (0.7)	2 (0.5)	0.744
	Congestive heart failure		13 (9.3)	31 (7.5)	0.314
	Cardiogenic shock		6 (4.3)	12 (2.9)	0.418
	Sustained ventricular tachycardia		5 (3.6)	22 (5.3)	0.413
	Stroke/TIA		1 (0.7)	1 (0.2)	0.441
	Permanent pacemaker		13 (9.4)	36 (9.2)	0.820
	ICD		2 (1.4)	8 (1.9)	0.703
	Death		18 (13.0)	26 (6.3)	0.017
	Acute kidney injury (n, %)		2 (1.4)	3 (0.7)	0.443
	Coronary-artery stenosis >50% (%)				
		Left main	12 (8.6)	40 (9.7)	0.774
		Left anterior descending	82 (59.0)	270 (65.4)	0.075
		Left circumflex	72 (51.8)	218 (52.8)	0.840
		Right coronary artery	95 (68.3)	329 (79.7)	0.409
Culprit vessel (%)					
	Left main		1 (0.7)	3 (0.7)	1.000
	Left anterior descending		10 (7)	27 (6.5)	0.789
	Circumflex		10 (7)	18 (6.5)	0.188
	Right coronary artery		81 (58.3)	289 (70)	0.015
	Saphenous graft bypass		1 (0.7)	4 (1)	0.789
	TIMI grade of culprit vessel before PCI				
		0	79 (56.8)	214 (51.8)	0.305
		1	7 (5)	42 (9.9)	0.066
		2	2 (1.4)	13 (3.1)	0.284
		3	9 (6.5)	61 (14.8)	0.011
	Number of coronary vessels with >50% lumen stenosis (%)				
		1	11 (7.9)	57 (13.8)	0.067
		2	29 (20.9)	106 (25.7)	0.137
		3 or more	46 (33.1)	182 (44.1)	0.023
	Discharge medications				
		Aspirin	121 (87)	387 (93.7)	0.898
		β -blocker	7 (5.0)	78 (18.9)	0.001
		Statin	112 (80.6)	358 (86.7)	0.319
		ACEI	88 (84.2)	293 (70.9)	0.864
		ARB	33 (23.7)	97 (23.5)	0.599
		Discharge Permanent pacemaker	7 (0.05)	6 (0.01)	0.024

TIA, transient ischaemic attack; ICD, implantable cardioverter-defibrillator; TIMI, thrombolysis in myocardial infarction; PCI, percutaneous coronary intervention; ACEI, angiotensin-converting enzyme inhibitor; ARB, angiotensin receptor blocker.

### 3.2 In-Hospital Deaths and Survival Postdischarge

**Fig. 5.**
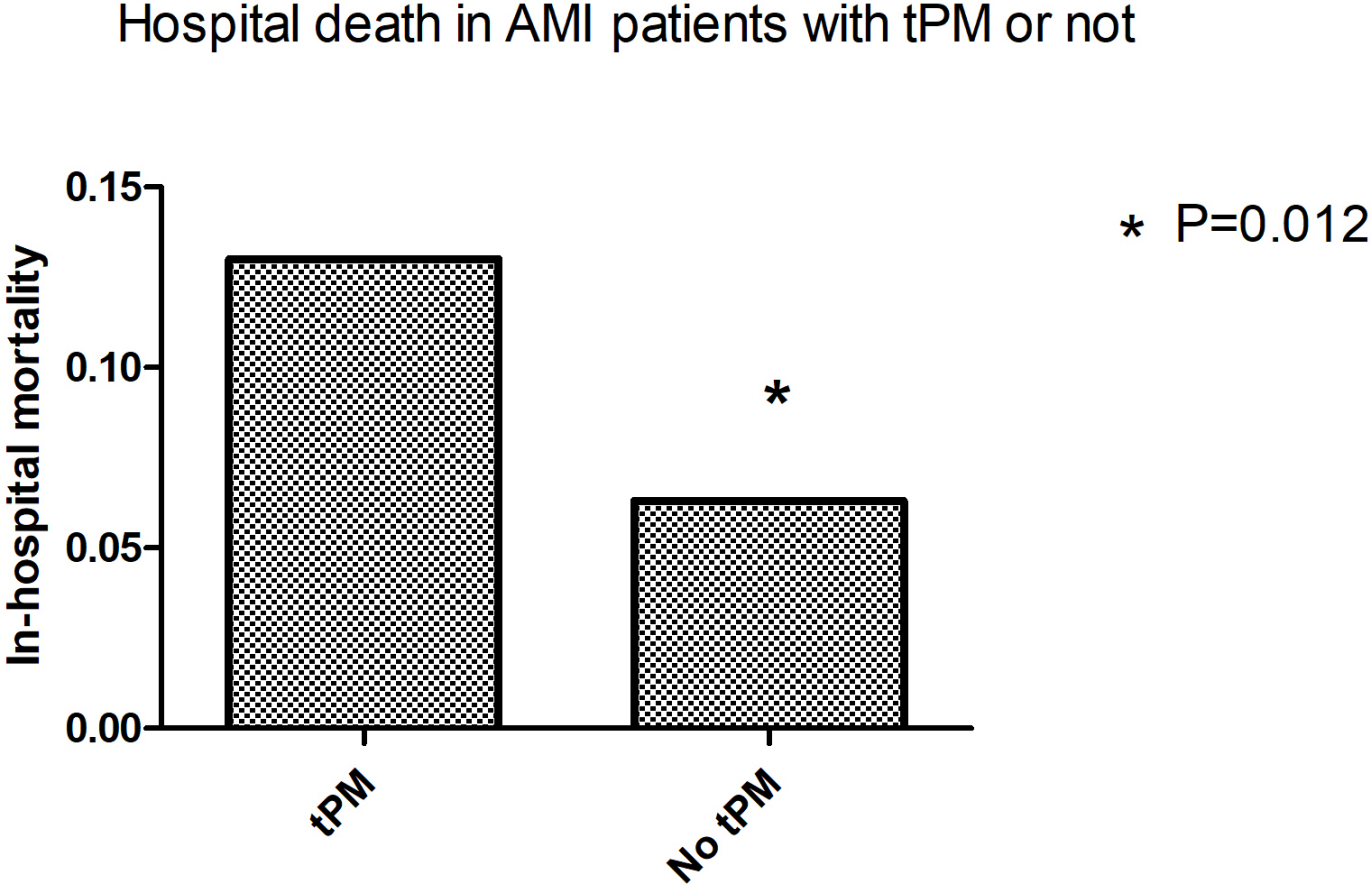
**In-hospital mortality in patients with AMI with or without tPM insertion**. tPM, temporary pacemaker; AMI, acute myocardial infarction.

**Fig. 6.**
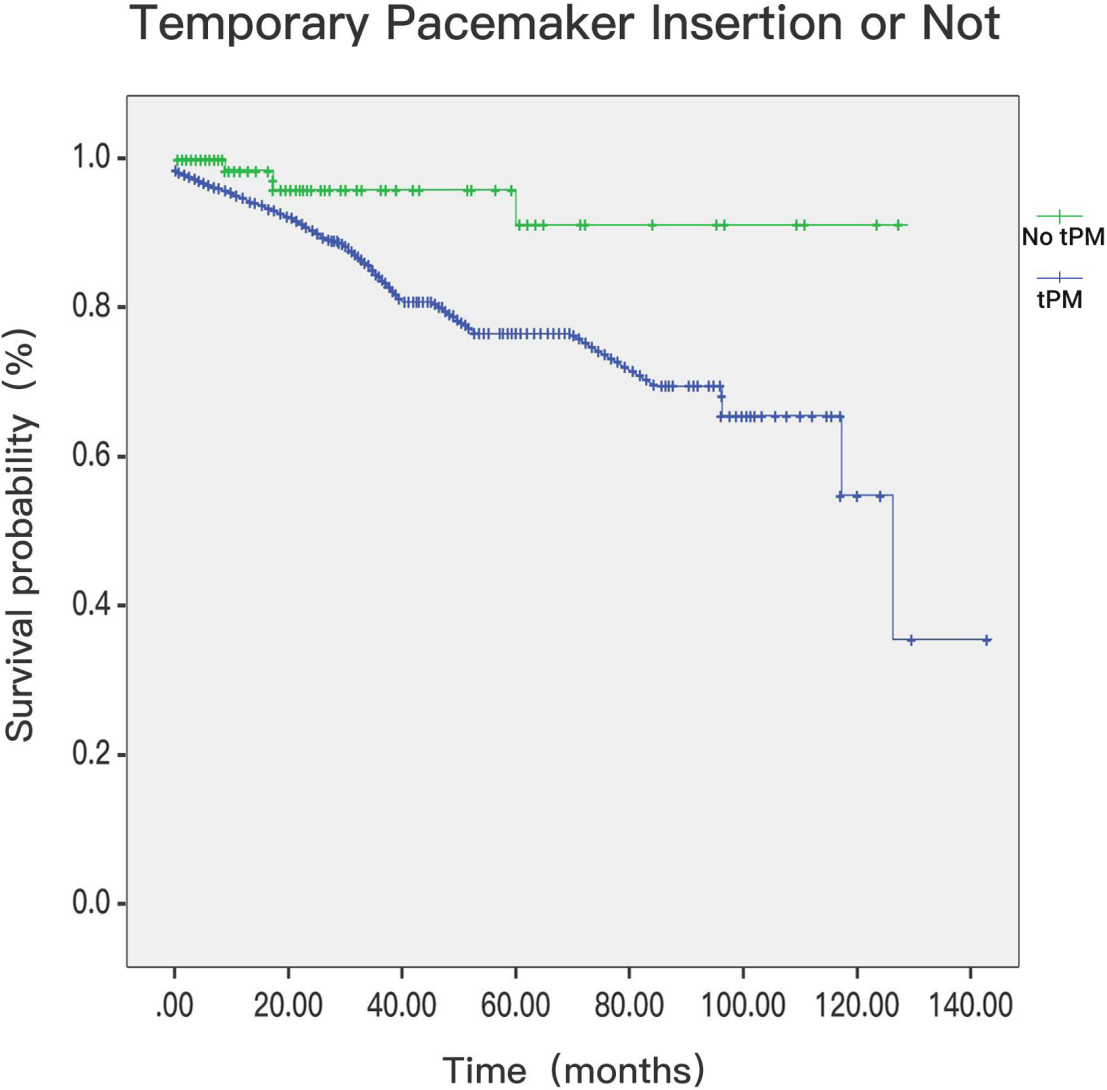
**In-hospital mortality in patients with tPM or not (*p* < 0.001)**. tPM, temporary pacemaker.

**Fig. 7.**
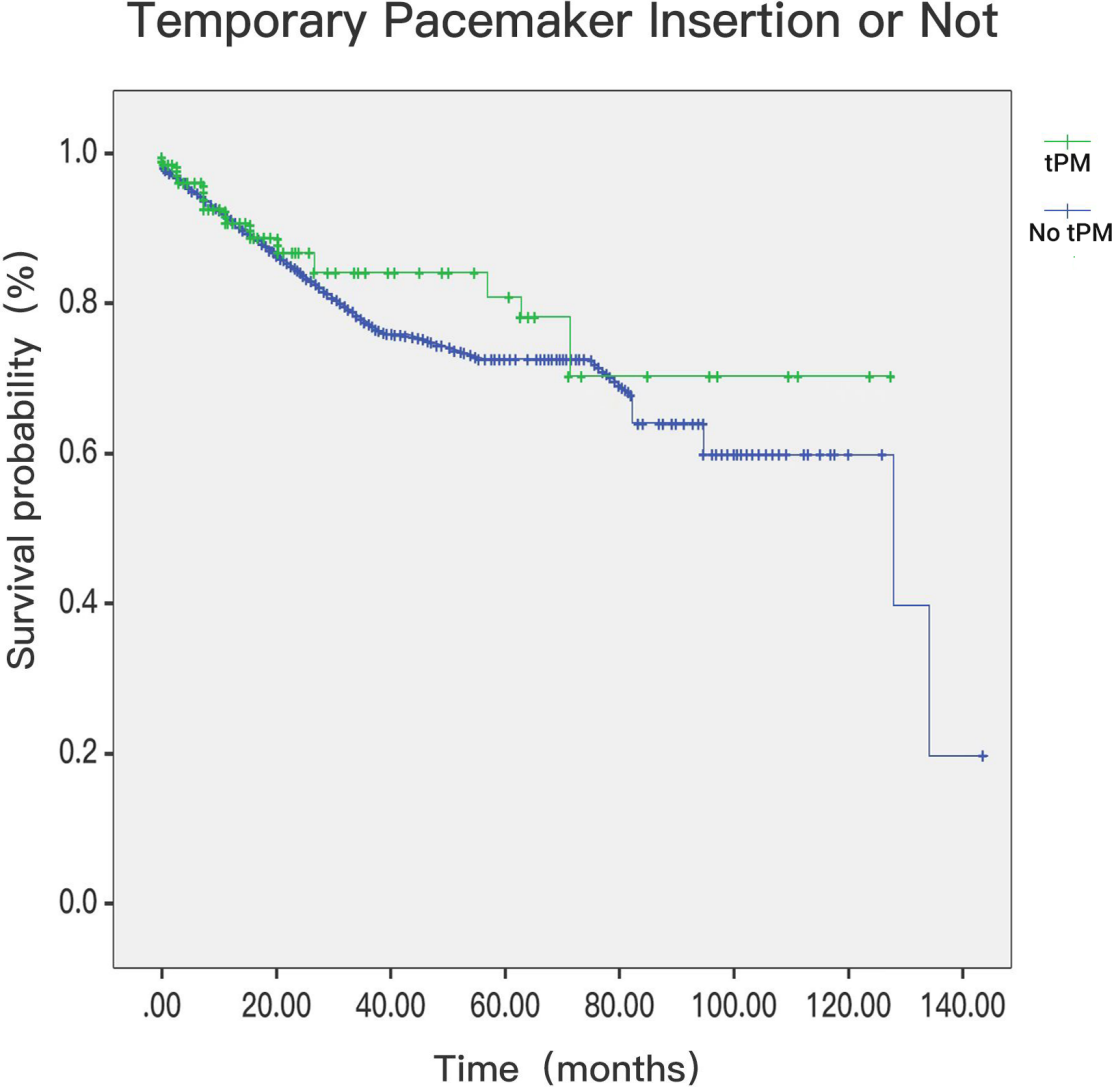
**Postdischarge mortality in patients with tPM or not (*p* = 0.006)**. tPM, temporary pacemaker.

However, for in-hospital mortality, 13.0% of patients with tPM implantationdied prior to hospital discharge compared with 6.3% without tPM insertion (*p* = 0.012, Fig. 5). After adjusting for three-vessel disease and successful percutaneous revascularization, this association also had statistically significant, with an odds ratio (OR) of in-hospital death of 4.0 (95% CI, 3.5–4.6; *p* < 0.001; Fig. 6). Of the 552 patients with cardiac ischaemia-associated arrythmia, follow-up was obtained for 97.1% of them. The patients with tPM insertion showed better postdischarge survival than patients without tPM insertion (log rank *p* = 0.006, Fig. 7) at a median follow-up period of 18 (7–45) months. The composite endpoints of modality and permanent pacemaker showed no significant difference between the tPM and non-tPM groups. The the secondary endpoints were in-hospital and post-discharge permanent pacemaker implantations. In-hospital permanent pacemaker showed no significant difference between the tPM and non-tPM groups (*p* = 0.820). Postdischarge permanent pacemaker implantation was more in tPM insertion group than non-tPM insertion group (*p* = 0.024). No significant difference in total mortality (both in-hospital and postdischarge) between the tPM and non-tPM groups was observed (15.8% vs. 18.9%, *p* = 0.418).

## 4. Discussion

This article describes the clinical characteristics and prognosis of patients with AMI complicated with arrhythmia for the first time. A total of 36,294 hospitalized patients with AMI in four medical centres over 10 years were analysed. Early studies showed that, before the era of intravenous thrombolysis and emergency PCI, AMI complicated with arrhythmia had a high in-hospital mortality rate. In 1970, Narva [2] found that the in-hospital mortality of patients with AMI complicated with high-degree AVB was 33%, and the proportion of tPM implantation in such patients was as high as 60%, which was much higher than that in the era of intravenous thrombolysis and emergency PCI. Our study found that, in the current era of vascular reconstruction, the in-hospital mortality of patients with AMI complicated with high-degree AVB was 8%, and the proportion of tPM implantation in patients with AMI complicated with arrhythmia (including sinus arrest, high-degree AVB and ventricular tachycardia storm) was 0.39%. In 1971, Rokseth [3] and others found that patients with AMI complicated with arrhythmia accounted for approximately 1.92% of patients, which was 1.37% compared with our study, indicating that the basic situation of AMI complicated with arrhythmia was no different from that in previous years.

In the last 10 years, the number of patients with high-degree AVB, tPM insertion, ventricular arrythmia storm, and in-hospital mortality has decreased year by year in the era of coronary artery revascularization. Our study confirms that, in the era of thrombolysis or emergency PCI for coronary artery revascularization, patients with AMI with arrhythmia had reductions in the critical rate of arrhythmia, decreases in the proportion of tPM insertion and reductions of in-hospital mortality due to coronary artery revascularization. These patients benefit from coronary revascularization, and this conclusion is consistent with the research of Hwang [4].

This study found that patients with AMI complicated with arrhythmia and tPM implantation had a high in-hospital mortality rate (*p* = 0.012). Temporary pacemaker implantation was possibly an independent risk factor for these patients. The results of recent studies [5, 6, 7, 8, 9] suggest that the in-hospital mortality rate of the tPM group is 2–5 times higher than that of the non-tPM group. Our study is consistent with these findings. Murphy [10] pointed out that the common complications of tPM implantation are ventricular fibrillation, myocardial perforation and sepsis. Our study found that there were significant differences in three-vessel disease, heart failure, cardiogenic shock, ejection fraction and ventricular tachycardia storm in the tPM group compared with those without tPMs, suggesting that the increased in-hospital mortality may be due to three-vessel disease, heart failure, cardiogenic shock and ventricular tachycardia storm. It is mainly the severity of myocardial infarction in patients with AMI complicated with arrhythmia, resulting in multiple vessels or larger infarct areas. The research results are consistent with those of Singh [1].

Our results showed for the first time that the postdischarge mortality of patients with AMI complicated with arrhythmia was lower in the tPM group. This finding is very interesting. It may be that patients with tPMs have ameliorated their arrhythmia in the hospital, and because of the severe condition of AMI, if emergency tPMs are inserted, the proportion of emergency revascularization is higher, resulting in a decrease in out-of-hospital mortality. This research result needs to be further researched because of the benefit of tPM implantation. Or does the increased proportion of revascularization in these patients reduce out-of-hospital mortality? Our results suggest that the left ventricular end diastolic diameter (50.4 ± 6.8 vs. 48.5 ± 7.1, *p* = 0.005) in the non-tPM group worse than that in the tPM group. Does it affect out-of-hospital mortality in the non-tPM group? Further research is needed to obtain the results of the impact guidelines. At the same time, we found that (Fig. 4), in patients with AMI complicated with arrhythmia, successful PCI within 24 hours is a protective factor for these patients and reduces in-hospital mortality, which is consistent with Sheldon [1] and other studies showing that receiving thrombolysis or PCI within 12 hours reduces the in-hospital mortality of these patients. Our study also found that the right crown is a culprit vessel, which is also a protective factor for patients with AMI complicated with arrhythmia. Perhaps due to the complexity and severity of vascular diseases, such as the left coronary artery or left main artery, the right coronary artery is a culprit vessel that is easily complicated with arrhythmia, which is easier to correct and reduces the in-hospital mortality of patients.

This study found for the first time that, in the tPM group, if the culprit vessel was the left main artery, whether revascularized or not, it was an independent risk factor. In the tPM group, Cardiogenic shock, acute renal injury and high BNP levels were independent risk factors for patients with AMI complicated with arrhythmia. In patients without tPMs, cardiogenic shock and anterior wall infarction are independent risk factors for AMI complicated with arrhythmia. These results are consistent with previous studies [11, 12] showing that acute renal injury and high BNP levels are independent risk factors for AMI.

In myocardial infarction combined with high-degree AVB or sinus arrest, the main mechanism is acute left ventricular inferior wall or posterior wall myocardial infarction, is caused by ischemia of the atrioventricular node artery and sinoatrial node artery, which occurs in the early stage of myocardial infarction, reflecting myocardial reperfusion, and activating parasympathetic nerves [13, 14, 15, 16]. In the subgroup analysis of our study, there was no significant difference in the proportion of tPM implantation in the group with myocardial infarction complicated with high-degree AVB or sinus arrest, indicating that this kind of arrhythmia may be more common in the early stage of myocardial infarction or it may be easily ameliorated by timely drug treatment or revascularization. Another subgroup analysis found that the proportion of patients with AMI complicated with ventricular tachycardia storm with tPM insertion was less than that without tPM insertion, indicating that revascularization is more effective and provides long-term benefit for patients with such arrhythmias in the era of emergency thrombolysis and PCI.

## 5. Conclusions

In the era of emergency thrombolysis or PCI, coronary revascularization can ameliorate the prognosis of patients with AMI complicated with various arrhythmias. Temporary pacemaker insertion in patients with AMI complicated with arrhythmia can reduce the postdischarge mortality of these patients.

## Data Availability

The datasets used and/or analyzed during the current study are available from the corresponding author on reasonable request.

## References

[1] Singh SM, FitzGerald G, Yan AT, Brieger D, Fox KAA, López-Sendón J (2015). High-grade atrioventricular block in acute coronary syndromes: insights from the Global Registry of Acute Coronary Events. *European Heart Journal*.

[2] Narvas RM, Kilgour JM, Basu SK (1970). Heart block in acute myocardial infarction: prognostic factors and role of transvenous catheter pacemaker. *Canadian Medical Association Journal*.

[3] Rokseth R, Hatle L (1971). Sinus arrest in acute myocardial infarction. *British Heart Journal*.

[4] Hwang YM, Kim C, Moon K (2016). Periprocedural temporary pacing in primary percutaneous coronary intervention for patients with acute inferior myocardial infarction. *Clinical Interventions in Aging*.

[5] Feigl D, Ashkenazy J, Kishon Y (1984). Early and late atrioventricular block in acute inferior myocardial infarction. *Journal of the American College of Cardiology*.

[6] Berger B, Ryan TJ (1990). Inferior myocardial infarction. High-risk subgroups. *Circulation*.

[7] Harpaz D, Behar S, Gottlieb S, Boyko V, Kishon Y, Eldar M (1999). Complete atrioventricular block complicating acute myocardial infarction in the thrombolytic era. SPRINT Study Group and the Israeli Thrombolytic Survey Group. Secondary Prevention Reinfarction Israeli Nifedipine Trial. *Journal of the American College of Cardiology*.

[8] Nguyen HL, Lessard D, Spencer FA, Yarzebski J, Zevallos JC, Gore JM (2008). Thirty-year trends (1975-2005) in the magnitude and hospital death rates associated with complete heart block in patients with acute myocardial infarction: a population-based perspective. *American Heart Journal*.

[9] Gang UJO, Hvelplund A, Pedersen S, Iversen A, Jøns C, Abildstrøm SZ (2012). High-degree atrioventricular block complicating ST-segment elevation myocardial infarction in the era of primary percutaneous coronary intervention. *Europace*.

[10] Murphy JJ (2001). Problems with temporary cardiac pacing. Expecting trainees in medicine to perform transvenous pacing is no longer acceptable. *British Medical Journal*.

[11] Feng Y, Wang Q, Chen G, Ye D, Xu W (2018). Impaired renal function and abnormal level of ferritin are independent risk factors of left ventricular aneurysm after acute myocardial infarction: A hospital-based case-control study. *Medicine*.

[12] Zhang Z, Guo J (2020). Predictive risk factors of early onset left ventricular aneurysm formation in patients with acute ST-elevation myocardial infarction. *Heart & Lung*.

[13] Simons GR, Sgarbossa E, Wagner G, Califf RM, Topol EJ, Natale A (1998). Atrioventricular and intraventricular conduction disorders in acute myocardial infarction: a reappraisal in the thrombolytic era. *Pacing and Clinical Electrophysiology*.

[14] Adgey AA, Geddes JS, Mulholland HC, Keegan DA, Pantridge JF (1968). Incidence, significance, and management of early bradyarrhythmia complicating acute myocardial infarction. *Lancet*.

[15] Wei JY, Markis JE, Malagold M, Braunwald E (1983). Cardiovascular reflexes stimulated by reperfusion of ischemic myocardium in acute myocardial infarction. *Circulation*.

[16] Koren G, Weiss AT, Ben-David Y, Hasin Y, Luria MH, Gotsman MS (1986). Bradycardia and hypotension following reperfusion with streptokinase (Bezold-Jarisch reflex): a sign of coronary thrombolysis and myocardial salvage. *American Heart Journal*.

